# Correlating Personal Resourcefulness and Psychomotor Skills: An Analysis of Stress, Visual Attention and Technical Metrics

**DOI:** 10.3390/s22030837

**Published:** 2022-01-22

**Authors:** Carmen Guzmán-García, Patricia Sánchez-González, Juan A. Sánchez Margallo, Nicola Snoriguzzi, José Castillo Rabazo, Francisco M. Sánchez Margallo, Enrique J. Gómez, Ignacio Oropesa

**Affiliations:** 1 Biomedical Engineering and Telemedicine Centre, ETSI Telecomunicación, Centre for Biomedical Technology, Universidad Politécnica de Madrid, 28040 Madrid, Spain; carmen.guzmang@upm.es (C.G.-G.); p.sanchez@upm.es (P.S.-G.); nicola.snoriguzzi@alumnos.upm.es (N.S.); enriquejavier.gomez@upm.es (E.J.G.); 2 Centro de Investigación Biomédica en Red en Bioingeniería, Biomateriales y Nanomedicina, 28029 Madrid, Spain; 3 Centro de Cirugía de Mínima Invasión Jesús Usón, 10071 Cáceres, Spain; jasanchez@ccmijesususon.com (J.A.S.M.); jcastillo@ccmijesususon.com (J.C.R.); msanchez@ccmijesususon.com (F.M.S.M.)

**Keywords:** stress, attention, personal resourcefulness, psychomotor skills, surgical skills, surgical education

## Abstract

Modern surgical education is focused on making use of the available technologies in order to train and assess surgical skill acquisition. Innovative technologies for the automatic, objective assessment of nontechnical skills are currently under research. The main aim of this study is to determine whether personal resourcefulness can be assessed by monitoring parameters that are related to stress and visual attention and whether there is a relation between these and psychomotor skills in surgical education. For this purpose, we implemented an application in order to monitor the electrocardiogram (ECG), galvanic skin response (GSR), gaze and performance of surgeons-in-training while performing a laparoscopic box-trainer task so as to obtain technical and personal resourcefulness’ metrics. Eight surgeons (6 nonexperts and 2 experts) completed the experiment. A total of 22 metrics were calculated (7 technical and 15 related to personal resourcefulness) per subject. The average values of these metrics in the presence of stressors were compared with those in their absence and depending on the participants’ expertise. The results show that both the mean normalized GSR signal and average surgical instrument’s acceleration change significantly when stressors are present. Additionally, the GSR and acceleration were found to be correlated, which indicates that there is a relation between psychomotor skills and personal resourcefulness.

## 1. Introduction

Modern surgical education is focused oTn making use of the available technologies in order to train surgical residents, monitor their progress and assess their surgical skill acquisition [1]. Surgical skills are, nowadays, commonly split into either technical or nontechnical categories [2]. Technical skills comprise a series of competencies, such as psychomotor skills, swift eye-hand coordination and dexterity [3]. Nontechnical skills encompass varied competencies such as cognitive readiness [4], decision making [5], interpersonal abilities (i.e., communication and teamwork [6,7]) and personal resourcefulness (i.e., attention and stress management) [2,8]. This artificial bifurcation has helped to further categorize surgical competency into subcategories of skills.

The applicable technologies for technical skills’ characterization (e.g., the instruments’ or hand’s motion tracking analysis) have been widely studied and effectively used in surgical educational environments [9,10,11,12,13]. Studies focusing on simulation have revealed that the objective assessment of psychomotor skills is feasible through the combination of motion-analysis-derived efficiency and quality metrics [14,15]. Efficiency metrics are related to measurable physical parameters, which makes them objective and reproducible (e.g., time, path length, speed, etc.). These metrics can be divided into motion-derived metrics (e.g., acceleration, economy of area, economy of volume, etc.) and force-derived metrics (e.g., the force applied in sutures or tissue handling). Quality metrics relate to a task’s definition and execution (e.g., number of errors, tissue damage, etc.).

However, as previously stated, surgical skills are not just technical in nature [16]. Nontechnical skills also play a major role when characterizing surgical skills; thus, it becomes necessary to develop automatic, objective monitoring and assessment methods for them. Innovative technologies are being developed, implemented and applied for the automatic, objective assessment of nontechnical skills [17]. For instance, visual attention and stress monitoring (especially based on wearable technologies) are gaining relevance within the surgical community due to their influences on personal resourcefulness [18].

Personal resourcefulness was initially defined by Brunckhorst et al. [19] as the ability to cope with stress and fatigue. Madani et al. [20] refined this definition by incorporating two main factors into it: (1) “self-awareness and metacognition” and (2) “management of modulators of attention, stress and goals”. Essentially, personal resourcefulness encompasses the ability that a surgeon must have to “self-regulate one’s cognitive processes and behaviours, with awareness of various modulating factors that can impact performance” [20]. Thus, personal resourcefulness includes maintaining focus for extensive periods of time amidst several distractions (e.g., sudden noises, surgical team movements, etc.), balancing attention between several intraoperative considerations (e.g., patient condition, performance of the surgical team, etc.) and managing the modulators of stress or attention [20,21].

### 1.1. Modulators of Attention

Visual attention and hand-eye coordination skills are essential for developing and mastering surgical techniques [22,23,24]. Different experimental studies have demonstrated a close relationship between the eye movements that are occurring during visual attention tasks and the way in which the information is processed at a cognitive level [25,26]. Some studies have shed light on the visual attention patterns between experts and nonexperts (based on the classification proposed by Land et al. to differentiate between experts and nonexperts [27], experts have more than 5 years of experience, while nonexperts have less than 5 years of experience), showing that, while experts tend to fix their attention on the target, nonexperts show rather erratic behavior, often focusing attention on their own instrument [22,23,24]. These studies suggest that a more efficient attention ability (i.e., the ability to shift the attention’s focus to areas requiring higher consideration) could give ground to other cognitive processes during surgical interventions. Given the importance of visual attention, it becomes essential to develop learning strategies to train and assess the skills that are related to visual attention and concentration. For instance, Wilson et al. [24] designed and validated the effectiveness of a surgical performance enhancement program that is based on gaze training by means of a measure of target locking (computed by subtracting the percentage of time spent fixating on the surgical tool from the time spent fixating on the target). Additionally, Richstone et al. [28] showed that eye metrics, such as pupil diameter, blink rates and fixation rates, could effectively differentiate between surgical experts and nonexperts (i.e., using linear discriminant analysis and neural network analysis) and, thus, might be used as an objective measurement of surgical skill.

Visual attention can also be used to train other surgical staff. Koh et al. [29] studied the differences in eye movements between expert and nonexpert nurses in cesarian section surgeries. Their study found that the expert nurses distributed their attention more effectively during high-stress stages of the surgery by better focusing on the important areas of interest for each stage. Areas of interest were defined by the distinct boundaries within which the scrub nurses perform their tasks depending on the stage of the surgery. Experts also exhibited increased performance (measured by means of Objective Structured Assessment of Technical Skills—OSATS—questionnaire). In a different experiment, Tomizawa et al. [30] examined the eye movements of perfusionists, perceiving that those with less experience visually inspected key information areas less often than the more experienced ones (who also distributed their attention more widely, exhibiting lower fixation rates). Additionally, gaze training has been demonstrated to enhance the acquisition of laparoscopic technical skills [24].

We hypothesize that attention-based training’s benefits can be increased by means of objective assessment methodologies. Currently, eye-tracking devices [31,32] and camera systems [33,34,35] (among other methods) are being used to monitor the surgeons’ visual attention, as well as their fatigue and concentration levels. However, none are being applied in order to bring additional information about the surgeons’ personal resourcefulness.

### 1.2. Modulators of Stress

There is evidence proving that intraoperative stress can affect the overall performance of surgeons (e.g., by complicating the communication and hampering the hand-eye coordination, as well as other motor functions) [36]. This may lead to inferior patient outcomes and bring about potential intra- and postsurgical complications [37]. Thus, the training of stress management skills can be said to have especial relevance, as surgeons will be more prepared to effectively manage stressful situations when encountering them.

Simulation-based training methods for stress management are used in the literature within laparoscopic training programs [38,39], specifically in box trainer- or mannequin-based tasks. Repeated simulation training in high fidelity settings [40] and the training of eye gaze under high-anxiety conditions [41] have also been used to train stress management. In addition, studies of mental training methods have demonstrated positive effects on participants’ stress experience and a reduction of their cognitive stress [42,43,44,45], as well as an improvement of their technical performance [39,44,46,47]. Finally, in the study by Lemaire et al. [48], a biofeedback-based stress management tool was designed and its effectiveness was demonstrated through a randomized control trial in which the mean stress score declined significantly for the intervention group. This tool monitors certain stress-related parameters (i.e., heart rate and blood pressure) and alerts surgeons when surpassing threshold levels so that they can apply their stress management techniques.

Based on the usefulness of this biofeedback-based stress management tool, numerous studies in the literature have focused on the characterization of stress in the surgical practice by means of simulation tasks (e.g., virtual reality or box trainers) including stressful situations (such as unexpected bleeding or sudden noises), so that residents are able to learn how to deal with such situations.

Due to the simplicity of its measurement, heart rate (HR) has been used in several studies via an electrocardiogram (ECG)-based stress-monitoring method [37,49,50,51]. HR has been demonstrated to increase when performing a surgery and even more so in laparoscopic surgery (as compared to open interventions) [50]. Some studies have also demonstrated the potential of HR to differentiate between expert and nonexpert surgeons [36]. In a study by Marrelli et al. [52], HR was found to be higher in nonexpert surgeons than in experts. They suggested that this might be an indicator of the expert surgeons’ enhanced stress management skills. Even though they recognize that the difficulty of the surgical procedure can influence cardiovascular alterations and cortisol levels, the way in which they are affected is significantly higher in junior surgeons than in senior surgeons.

Other ECG-derived metrics are related with HR variability (HRV), which is defined as the variation in the interval of time between the heart beats. Weenk et al. [53] and Grantcharov et al. [54], in their respective studies, evaluated how the standard deviation of the interval between two heart beats (SDNN) and the square root of the mean interval between two heart beats (RMSSD) were affected by stress levels during surgical performance. Both concluded that these two metrics decreased significantly during laparoscopic surgical procedures.

Typical HRV-derived metrics in the frequency domain that are used in surgical learning environments are related to the power in two bands: low frequency (LF) (range 0.05–0.15 Hz [55,56]) and high frequency (HF) (range 0.16–0.45 Hz [56]). This distinction is of particular importance since LF is commonly associated with the activity of the sympathetic nervous system (which triggers the stress response) [57]. Thus, the most popular frequency-based metric is the ratio between the absolute power of the signal in the low and high frequency bands (LF/HF). This ratio has been demonstrated to increase significantly when presented with stressful situations (such as surgical procedures) [50,53].

Despite the close relation observed between HR and HRV with stress, this monitoring method can be influenced by other factors since it measures both the stress response and HR fluctuations of the individual that are not necessarily related to stress (e.g., caffeine intake, arrhythmias, etc.). This is why additional monitoring methods are usually coupled with the analysis of ECG-derived metrics.

For instance, measurement of the electrodermal activity or the use of the galvanic skin response (GSR) have been reported in surgical educational environments for stress monitoring. The potential obtrusion to the normal performance of the task when using GSR electrodes (usually placed in the fingertips) can be considered a major drawback. However, new electrodes have been developed and are being improved in order to measure the GSR via a wrist-band [58].

As an example of the use of the GSR in surgical environments, Smith et al. [59] proposed a workstation to monitor surgeons’ mental workload that is associated with surgical procedures (i.e., the amount of mental resources that are required to perform a set of concurrent tasks [60]) with their GSR as an indicator of their mental stress levels while performing a suturing task. The data that were obtained for this parameter were correlated to self-rated levels of stress during the task, resulting in an accurate characterization of stress levels in surgical contexts (it was found that GSR increases when stress does). In the experiment of Ershad et al. [61], experts showed lower variability in the GSR signal, while nonexperts showed higher variability.

The metrics that are related to stress that can be extracted from GSR measurements that are found in the literature are varied in their complexity. The most popular approaches are mainly descriptive (i.e., the mean, standard deviation and sum of the GSR signal). For instance, Hurley et al. [62] used these metrics to find the differences in the stress levels between standard laparoscopic surgery and robotic surgery. These metrics have also been used to validate their relation with other variables, specifically with workload [63] and performance [64].

A different approach consists of analysing the peaks of the GSR signal. An example of this is the study by Wilson et al. [65] in which they discovered that the mean peak values are correlated with the difficulty of the task and that the nonexpert fellows presented mean peak values that were higher than those of the expert fellows.

A more complex processing of the GSR signal was proposed by Bakker et al. [66], who used a solution consisting of an aggregation step in the time axis and a discretization step for the GSR signal. The aggregation in time was performed by using the overlapping time windows to segment the signal and extract the mean values for each segment. With the discretization, only a finite number of the GSR values were considered. This approach has been found to be more robust and reliable in the presence of noise and artifacts; but, being that the processing is more complex, it is not much used in the literature [67].

Besides ECG and GSR, other monitoring systems have been used in order to monitor stress in a surgical educational context, though their use is less frequent, mainly due to their higher intrusiveness. For instance, in the study by Bartolomeo et al. [68], both HR and surface electromyography (EMG) were simultaneously measured by electrodes that were located on the left and right upper trapezium. They proved that muscle activation is much higher in live procedures than in virtual environments.

Electroencephalography (EEG) has seldom been used as a stress monitoring method in the surgical educational context, mostly due to the intrusive nature of EEG and the difficulty in the stress characterization in a specific frequency band. An example of the metrics that can be extracted from EEG in order to monitor stress in surgical environments is the ratio of the beta band over the alpha band. Duru et al. [69] designed an experiment in order to monitor the mental workload during a simple laparoscopic nephrectomy using this metric, effectively characterizing the most difficult and stressful phases in the intervention. Meanwhile, Morales et al. proved that higher stress levels and surgical complexity are associated with higher activity in the beta-frequency of the EEG [70].

Lastly, functional near-infrared spectroscopy (fNIRS) has gained acceptance as a stress-monitoring method due to its applications in cognitive and behavioral studies [71,72]. fNIRS is an indicator of the activity of the prefrontal cortex (PFC), which is activated (among other cases) when presented with stressors. Specifically in surgical training environments, Shetty et al. demonstrated that nonexperts had higher levels of PFC activity than experts [73].

Other studies have focused on the assessment of stress during surgical procedures based on standard scales, such as the State–Trait Anxiety Inventory (STAI) [74,75,76,77] or ad hoc scales for surgical interventions [38,78]. Some studies have used the STAI as a correlation method for comparison against the physiological indicators of stress [38,75,76,77].

For instance, Arora et al. [75,79] designed a stress assessment tool (the imperial stress assessment tool—ISAT), by which quantitative data (HR and cortisol levels) and qualitative data (stress self-assessment by means of the STAI test) were gathered. ISAT was validated by scheduling 54 procedures varied in complexity (and thus, the level of stress that was induced), with the same 11 surgeons. The results of their validation indicated that both STAI and HR were higher for stressful procedures as compared to non-stressful ones. This suggests that both are highly correlated to the stress response. In another study, they added technical performance measurements [74] by using a MIST-VR simulator [80] by which the path length, total simulation time and number of errors could be derived. Both STAI and HR correlated with the performance measurements.

### 1.3. Goals of This Study

As observed in the literature, there are numerous studies that have focused on the characterization of stress and visual attention in surgical educational environments. Furthermore, it has been demonstrated that the application of monitoring technologies characterizing stress can be used to train personal resourcefulness (i.e., to recognize and manage modulators of stress and attention).

The tasks that are used for personal resourcefulness training are greatly varied and depend on the specific learning objective as well as the goals of the study itself. For instance, Platte et al. [81] used the valid and reliable star-track test [82] mounted on a box trainer to measure surgical performance while presented with the Norinder arithmetic test (as a stress-triggering method) in order to look for differences in performance between stressful and non-stressful situations in terms of the number of errors and the amount of time that was spent on the task. In the Norinder test, participants are required to add and subtract a series of paired one or two digit numbers under a certain time threshold. The Norinder arithmetic test has been demonstrated to trigger a stress response in subjects [83,84]. The results indicated that there were indeed differences. However, the individuals’ stress was not quantitatively assessed (i.e., the physiological parameters that are correlated to the stress response were not monitored), which does not allow effective demonstration that the stress response was triggered during the task.

In addition, few studies have explored the potential of monitoring parameters for the objective and quantitative assessment of personal resourcefulness or whether these two skills are related to one another (i.e., whether the stress- and attention-based parameters may be related with other surgical skills metrics, such as the use of motion-analysis-based parameters to characterize psychomotor skills).

Thus, this present study has two main goals: (1) to quantitatively characterize the stress response during a basic surgical training task, and (2) to find whether the metrics that are related to psychomotor skills and personal resourcefulness in surgical education are correlated.

To verify or reject these hypotheses, we present an analysis of metrics that were derived from physiological parameters related to stress, gaze and laparoscopic instrument tracking in order to study how they are influenced by stressors and how they influence one another.

## 2. Materials and Methods

### 2.1. Subjects

Laparoscopic surgeons collaborating with the Centro de Cirugía de Mínima Invasión Jesús Usón (Cáceres, Spain) participated in the study after providing informed consent. Demographic information was gathered from each participant (i.e., age, gender and experience level). The participants were grouped, according to their years of previous surgical training experience, into experts (>5 years of experience) and nonexperts (<5 years of experience) [27].

The participants did not take part in any vigorous activity within 30 min of the task performance in order to avoid a bias in the ECG measurements. The exclusion criteria included concurrent use of medications affecting heart rate.

### 2.2. Experimental Setup

**Figure 1:**
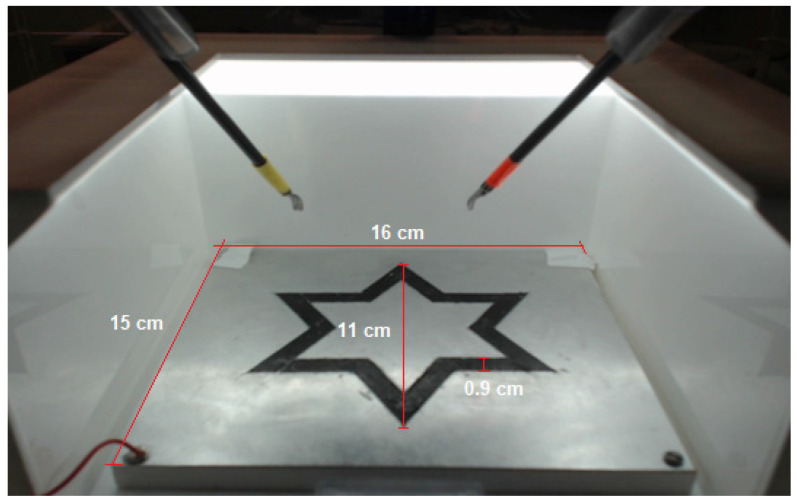
Star-track test (as seen in the box trainer).

In order to allow the participants to engage their personal resources (i.e., to recognize and manage modulators of stress and attention), a specific task that was based on the star-track test [82] (see Figure 1) and the Norinder test was carried out. This combination was selected in light of the results that were obtained by Platte et al. [81] in an attempt to confirm their results via the quantitative assessment of stress. The addition of the Norinder test, in which stress and divided attention are triggered, assesses personal resourcefulness (since it includes the management of modulators of stress and attention). Furthermore, the cognitive demands that are necessary to solve the Norinder test can be associated with those of real surgical environments (only to a lesser extent) [85].

The star-track test consists of following a star pattern with the laparoscopic instrument within a box trainer (using the dominant hand), as efficiently and quickly as possible and within the star’s borders [81]. More specifically, the star pattern was created using an aluminum plaque (16 × 15 cm), on top of which a 0.9 cm-wide strip of duct tape was placed, forming a star pattern (point to point distance: 11 cm).

None of the participants were familiarized with the task nor allowed to practice it beforehand. For the first phase of the experiment, the participants were asked to complete the star-track test and reminded to keep the instrument in continuous contact with the plaque. This had to be done a total of 10 times: 5 times counterclockwise and 5 times clockwise, as defined in the study by Platte et al. [81].

The second phase of the test was identical to the first but it included the Norinder arithmetic test and was carried out immediately after the first phase. The participants were encouraged to do the Norinder test with as few errors as possible. The test was always the same for all of the participants. The mathematical questions of the Norinder test had to be solved in under 5 s. When answered (or if not answered in time) the next question was shown. The length of the test (i.e., the number of additions and/or subtractions that were presented) depended on the time that the participant took to complete the 10 laps of the star-track test. The Norinder test was presented on a different screen from the main one on which the video feed from the inside of the box trainer was shown and each question was read out loud by the research assistant. This slightly diverted the attention of the surgeons while triggering the stress response. The results of the Norinder test (as mentally calculated by the participants) were verbally stated by the participants and manually introduced (via keyboard) by the research assistant. They were automatically saved in a text file and then compared with the actual answer in order to avoid participants randomly answering them. Random answers would indicate that no stress was triggered because no actual mental calculations were taking place and these results would be discarded from the analysis. In order to evaluate for randomness, the participants’ answers were compared with the correct answers. If few questions were answered correctly (less than 15%), it was concluded that the answers were given at random and, as a result of this, the data were discarded.

Additionally, the level of stress that was perceived by the participants was collected on a Likert scale from 1 to 5 (1 being not stressed at all and 5 being very stressed) both before the experiment and after its completion.

### 2.3. Monitoring Application Description

**Figure 2:**
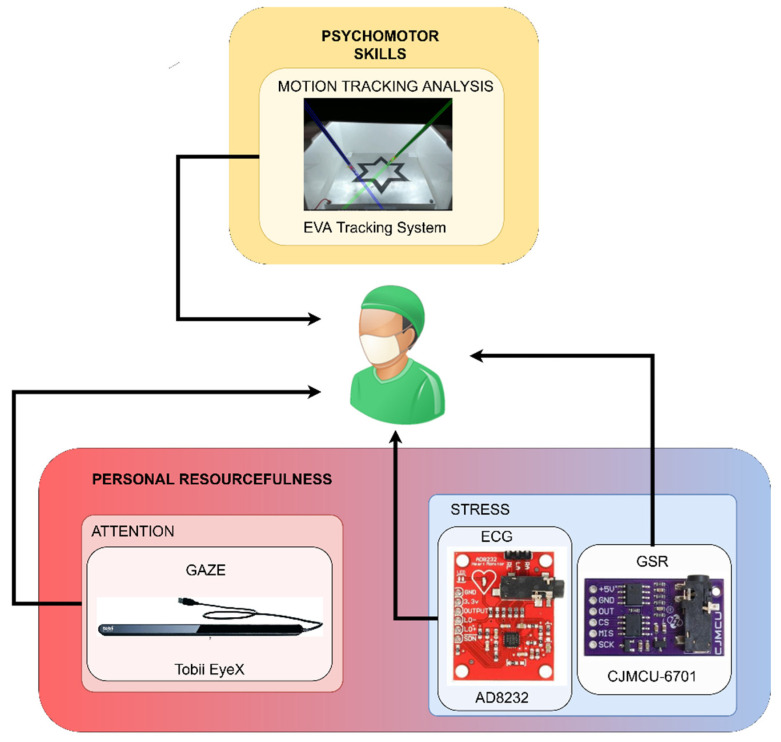
Monitoring parameters in the designed experiment.

For the experiment, a combination of the (1) physiological signals related to stress (i.e., ECG and GSR), (2) gaze coordinates of the participants on the screen that were related to visual attention and (3) motion analysis parameters (MAPs) that were obtained from the tracking of the dominant laparoscopic instrument was obtained (Figure 2).

#### 2.3.1. Stress Module

**Figure 3:**
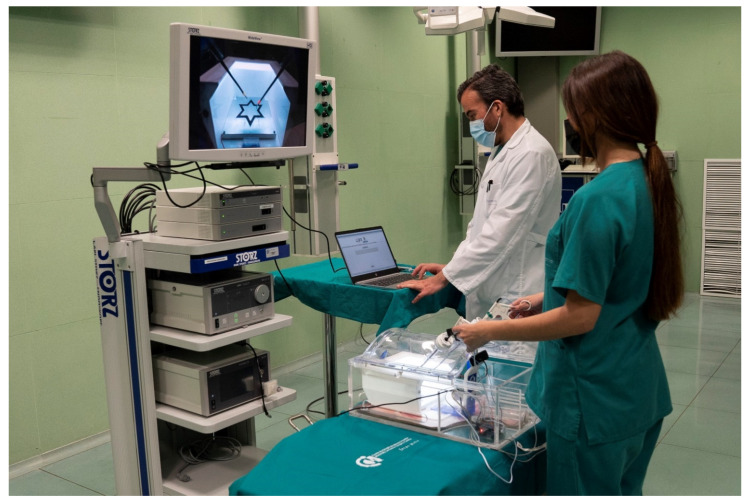
Setup of the experiment. ECG and GSR sensors are located inside the box trainer (red light). Eye tracker located immediately below the monitor showing the video feed captured by the camera inside the box trainer. The laptop is running the application gathering data. Research assistant is ready to start the second phase of the experiment, in which he will read mathematical questions out loud.

To monitor stress, we used ECG and GSR commercial measurement devices in order to measure the HR and sweat gland activity, respectively. These two physiological signals were selected due to their growing acceptance and accuracy [36,86] as well as their demonstrated reliability in preliminary tests in surgical settings. The incorporation of EMG to measure muscular activity in the trapezius was originally considered. Contrary to the ECG and GSR, the EMG was discarded after the preliminary tests revealed no response towards the math problem-based stressor. Additionally, both the ECG and GSR were not intrusive for surgeons (unlike an EEG, for instance). More to the point, the electrodes were located in order to ensure that the cables did not interfere with the normal performance of the surgeon (Figure 3).

The devices were integrated on a Wemos D1 mini microprocessor with a sampling rate of 100 Hz. The total sample size depended on the time that was taken to complete the experiment, with an average of 289,000 samples having been yielded per subject. The sensor that was used to monitor the ECG was the AD8232 (SparkFun, Niwot, CO, USA). It consists of an integrated signal conditioning block for ECG [87] that is designed to extract, amplify and filter the small ECG signal in the presence of noisy conditions, such as muscle motion. To monitor the GSR, the CJMCU-6701 (DIY Electronics, Durban, KZN, SA, South Africa) [88], a signal conditioning block for GSR, was used. Both of the sensors were connected to different pins of the Wemos D1 mini microprocessor.

To measure the ECG, snap wet gel electrodes were used since the amount of the electrode’s surface that is in contact with the skin is higher, thus providing a more accurate reading. The ECG electrodes were located under the right and left pectoral muscles and immediately below the rib cage [89]. These locations were selected due to the reduced amount of movement artifacts that are caused by muscular activity. The electrodes that were used to measure the GSR were located in the proximal phalanx of the index and middle fingers in order to achieve valid values.

The raw signal that was obtained by the ECG sensor was processed in order to obtain metrics related to the HRV and to the frequency-domain. More concretely, the signal was first filtered by means of a Butterworth high-pass filter (order = 3, F_c_ = 20 Hz) in order to remove the noise or distortion that was present in the signal. Baseline variations (i.e., respiratory noise, mean value due to electrodes contact, etc.) were then removed by means of an asymmetric least squares smoothing. This method uses a smoother with an asymmetric weighting of the deviations in order to get a baseline estimator. In doing so, the baseline can be removed while retaining the signal peak information. The resulting filtered signal was standardized (i.e., the values of the filtered signal were subtracted by the mean of the complete signal and divided by its standard deviation). After this, the local maxima were found (i.e., the peaks corresponding to the QRS complexes) by comparing neighboring amplitudes.

Once the QRS complexes were found, the NN intervals were computed by finding the distance between the peaks. The HRV signal was derived from the NN intervals and the time between them. The fast Fourier transform was applied to the interpolated HRV signal in order to select the high and low frequency components. The metrics that were used in this study were calculated according to the formulas that are shown in Table 1.

The raw signal that was obtained by the GSR sensor was normalized in order for it to be compared between the participants. Only the mean values were calculated as a metric of the GSR, since other validated metrics were not relevant for the specific data of the proposed experiment. More specifically, the slope was discarded after analysing each individual GSR signal and finding no common pattern in the slope, while peak analysis seemed irrelevant since the stress was continuous along the experiment instead of having been inflicted at given instants.

#### 2.3.2. Attention Module

To monitor attention, we used the commercial eye-tracker Tobii EyeX (Tobii AB, Stockholm, Sweden). Tobii EyeX is an eye-tracking device that uses infrared light to track the pupil’s motion, even when wearing glasses. It is placed at the bottom of the screen and can track the eye movement, the eye position and the gaze (i.e., the point in the screen that the person is looking at). In our particular case, we only used the gaze coordinates in order to compute the metrics to be correlated to personal resourcefulness. Before starting the experiment, the eye tracker was calibrated for each participant. To do this, they were asked to follow, with their gaze, a series of dots that were shown on the screen. The calibration tool (which is incorporated within Tobii’s software, version 2.16.5) automatically fitted the parameters for better recognition of the participant’s pupils.

**Figure 4:**
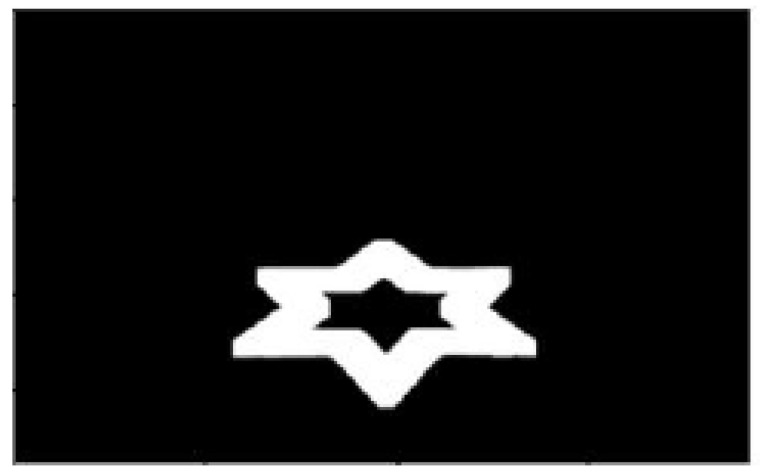
Example of the ROI that was used to extract the ratio of gaze points.

The visual attention metrics that were extracted from the eye-tracker were the fixation rate (i.e., the rate at which at least two gaze points were at a maximum distance of 30 pixels apart), the average fixation duration (i.e., the amount of time a fixation was given) and the gaze ratio inside a region of interest (ROI)—namely the star pattern that was to be followed (Figure 4).

#### 2.3.3. Performance Module

To monitor performance we used the Endoscopic Video Analysis (EVA) tracking system [12]. The EVA system enables the recording of the laparoscopic instruments’ motion based on a nonobtrusive video tracking algorithm. Specifically, the EVA system employs information about the laparoscopic instruments’ shaft edges in the image, their insertion point and the camera’s optical center in order to track the 3D position of the instrument’s tip. Thus, the system allows the analysis of the instruments’ movements and renders a set of MAPs for the effective assessment of surgical psychomotor skills using the information that is obtained from a single camera. These MAPs (Table 2) were used to analyze the correlation of psychomotor skills with personal resourcefulness.

Calibration of the EVA Tracking System had to be done beforehand using the video that was recorded during the experiment. This calibration essentially consisted of selecting the appropriate values in order to ensure that the EVA algorithm only recognized the marker of each laparoscopic instrument. This was manually done for each of the videos that were recorded during the experiment. Once the calibration was achieved, the corresponding video was fed to the EVA system for its coordinate and MAPs extraction. The MAPs were automatically calculated and uploaded to MongoDB (MongoDB Inc., New York, NY, USA) [90].

### 2.4. Data Analysis

To test for normality, the Shapiro–Wilk test was used for all of the metrics. In addition, QQ plots for all of the metrics were analyzed in order to ensure that the distribution was indeed normal or not normal (to account for the low sample size). Descriptive statistics for each of the participants are presented as a mean with standard deviation (SD) or a median with range, depending on the normality of the data’s distribution. The values of the MAPs, gaze analysis and physiological metrics of the first phase of the experiment were compared with those of the second phase using the ANOVA or Mann–Whitney test, depending on the normality of the data. These were done in order to analyze whether there were significant differences in the presence of stressors during the laparoscopic practice; thus, to know if the analyzed metrics were a good indicator of stress.

Additionally, a correlation analysis was performed, according to either Pearson’s or Spearman’s correlation coefficient, depending on the normality of the data, in order to find correlations between the psychomotor skills and personal resourcefulness skills metrics.

We adopted a critical α level of 0.05 to keep false discoveries at a traditional level as we sought to improve the reliability and effect size of the tests for the hypothesis. We used the Benjamini–Hochberg [91] method in order to provide a false detection error rate correction.

All of the statistical tests were carried out using RStudio 1.3.1093 (RStudio, Inc., Boston, MA, USA).

Lastly, we conducted a post hoc power analysis using G*Power [92,93] in order to find out whether the experiment had enough power to (1) detect the differences in the parameters under the stressors’ effects and (2) correlate the parameters of personal resourcefulness and psychomotor skills.

## 3. Results

In the initial experimental design, 20 surgeons were to be recruited. However, due to COVID-19, these numbers were reduced, mainly due to the unavailability of the surgeons. Thus, we recruited a total of 13 surgeons. However, due to unforeseen technical issues of the application recording the data (i.e., USB disconnection mid-task), some of the data from the stressful phase from 5 surgeons were not correctly recorded and were not included in the analysis. Therefore, eight surgeons were included in this study, (4 males and 4 females, aged 32 ± 5), following the protocol that was described in Section 2.1.

Two of the participants were considered experts (>5 years of experience). The remaining six participants were nonexperts. The mean perceived stress levels (±SD) before their participation in the experiment (on a 5-point Likert scale) were 1.423 (±0.847) whereas, by the end of the experiment, they were 3.975 (±0.814), increasing by 179% (*p* < 0.001). The analysis of the answers to the mathematical questions revealed no random patterns; thus, the data from all of the participants were included in the study.

**Figure 5:**
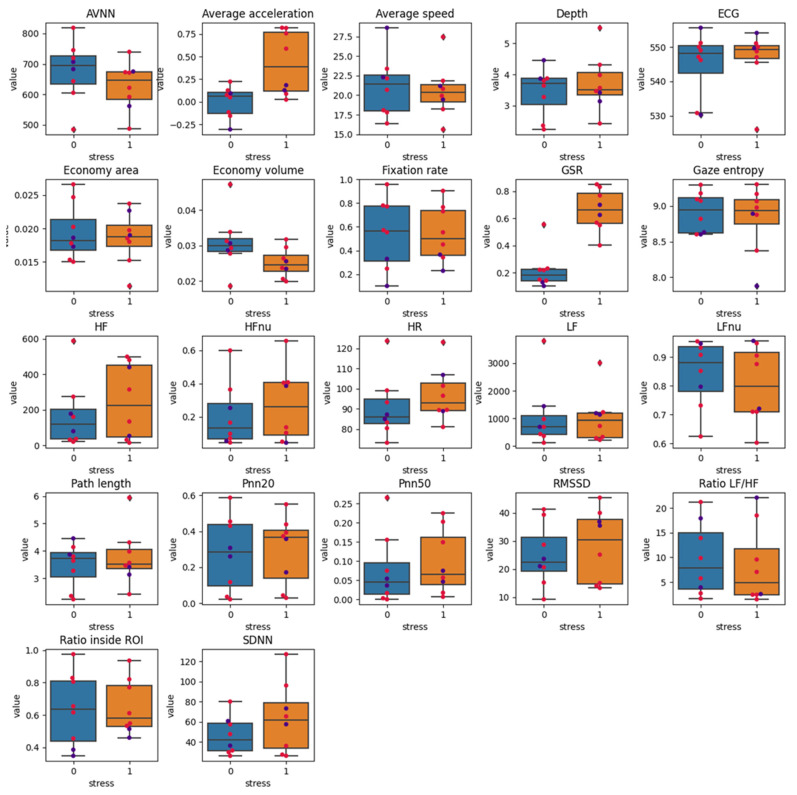
Box plot of phases 1 and 2 of the experiment for each of the metrics. Red and blue dots indicate the value for non-experts and experts, respectively.

The average speed of acceleration significantly increased by 15.09% in the stressful phase of the experiment as compared to the first phase (*p* < 0.05) and the GSR increased by 261% (*p* < 0.005) (Figure 5). The post hoc power analysis indicated that the effect size that was obtained for the average acceleration was 1.59 and 2.97 for the GSR (these are large effects, according to Cohen’s effect size conventions [94]). The powers that are required to detect effects of this size were determined to be 0.819 and 0.999, respectively. The remaining metrics did not present significant differences between the experimental phases. For further information, please see Table S1 in the Supplementary Materials.

The correlation analysis suggests that the GSR scores are correlated with the subjective stress that was stated by the participants at the beginning and end of the experiment (ρ = 0.79, *p* = 9.99 × 10^−4^). The GSR was also shown to correlate with the average acceleration (ρ = 0.82, *p* = 2.017 × 10^−6^). The post-hoc power analysis showed that the power for the correlation between the GSR and the subjective stress level is 0.99 (effect size = 0.88); and between the GSR and acceleration is 1 (effect size = 0.9).

**Figure 6:**
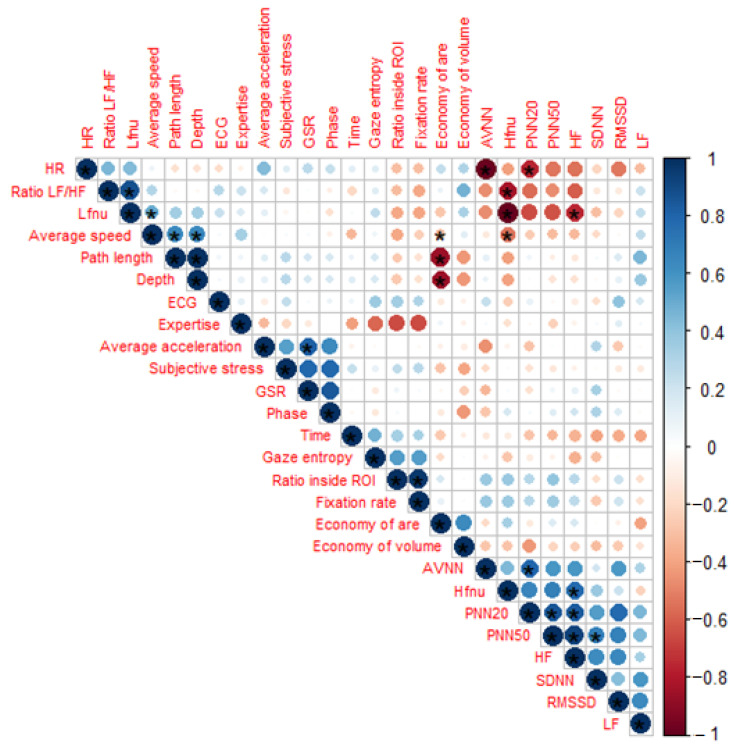
Correlation matrix. Color map and size represent the correlation between the variables. A star is depicted whenever the correlation was statistically significant according to Pearson or Spearman’s test.

The complete correlation results are reported in Figure 6.

## 4. Discussion

In this study, we analyzed 22 different metrics that were obtained during laparoscopic practice: 12 of which were derived from the ECG (in relation with stress), 1 of which was derived from the GSR (in relation with stress), 3 of which were derived from gaze analysis (in relation with visual attention) and 7 of which were derived from the motion tracking analysis of the dominant laparoscopic instrument (in relation with psychomotor skills). Specifically, the analysis was aimed at determining whether (1) stress could be characterized through these metrics, and (2) there was a relation between the metrics of psychomotor skills and personal resourcefulness.

The normalized GSR average value was found to be the most relevant parameter in terms of its relationship with stress, with an increase of 261% between the baseline and stressful phases of the experiment and a high, significantly positive correlation with the subjective stress rating that was stated by the participants. The relevance of the GSR average values has already been demonstrated in the literature and appears as one of the trending parameters for measuring stress in surgical environments [59,60,61,62,63,64,65,66,67].

Although the literature suggests that the metrics that are derived from ECG and HRV analyses are highly correlated with stress, none of the time- or frequency-derived metrics seem to have captured the variations in the HR as expected. However, we could not find evidence on how these metrics change with stress (i.e., not all of the subjects showed an increase or decrease in these metrics). This may be due to the fact that a baseline level was not established before the performance of the experiment. This may have led to unwanted bias in the results; for instance, the consumption of caffeine may have led to differences in HR [95].

The average acceleration of the surgical instrument increased significantly during the stressful phase of the experiment (by 15.09%). However, the overall time did not decrease significantly, which suggests that the movements of the instruments in the stressful phase were more abrupt than in the previous phase of the experiment.

The fact that we were able to correlate the GSR (which is a stress parameter) to acceleration indicates that there is a relation between psychomotor skills and personal resourcefulness skills. Other studies have correlated basic psychomotor skills metrics (e.g., the number of errors, time of completion, etc.) with stress parameters through subjective stress scales (e.g., STAI, NASA-TLX). We were able to do so in a quantitative manner and using technical metrics (i.e., acceleration). However, since only the GSR was found to be correlated to stress and, in light of the low power that was yielded in the case of ECG-derived metrics, further studies are necessary to confirm or refute this hypothesis.

The reason why we selected the initial sample size is partly based on the numbers used in similar studies (e.g., Tomizawa et al. [30] used the data from 4 participants and Jones et al. [50] used the data from 6 participants), which are generally low due to the difficulties in the recruitment of surgeons (and even more so in the case of expert surgeons), essentially based on their tight schedules. However, the aftermath of COVID-19 reduced surgeons’ availability even more and the safety limitations that were imposed made it difficult to recruit more surgeons. Thus, we ended up with a lower number of surgeons than initially calculated, which turned out to be the greatest limitation of this study.

It is due to this low number of surgeons that we could not compute an in-depth statistical analysis in order to find differences between the experts and nonexperts. Nevertheless, we did carry out a descriptive analysis, finding that, during the stressful phase, two of the three metrics that were derived from the gaze analysis were found to be higher in the nonexpert surgeons as compared to the experts during the stressful phase of the experiment (i.e., the ratio of fixations inside the ROI and the fixation rate). This is consistent with the literature, where fixation analysis has demonstrated to be in close relation to surgical expertise (and even suggested as a possible means towards training) [22,96,97]. This may be indicative of a lower cognitive load in the experts; due to the internalization of knowledge and surgical practice, experts are able to focus not only on the main surgical area but on other areas of the field of view. However, recognizing the low number of experts that were included in this study as one of its greatest limitations, we propose to further explore this in future research with higher participation and complete statistical analysis.

Additionally, none of the psychomotor metrics were found to be influenced by expertise. This may be due to the simplicity of the task (i.e., the task was so simple that there were little to no variations between experts and nonexperts) [98]. Thus, implementing a more difficult laparoscopic task in order to increase the cognitive load (such as suturing) while including stressors could lead to greater variations and better characterization of the differences between expertise levels using the same metrics as in this experiment. This could also be due to the lack of participants to compare experts and nonexperts, for which we highlight again the importance of conducting further experiments with more participants, once proven feasible that the measurements are valid and that some of the parameters that were monitored effectively measure surgical stress (i.e., the GSR and acceleration).

## 5. Conclusions

This study stems from the need to find objective assessment methodologies for personal resourcefulness skills in surgical education, while researching the relation between psychomotor and personal resourcefulness skills. To address this need, we proposed to analyze the influences between a set of metrics that are related to stress, gaze and instrument position and how they are affected by stressors and expertise.

After analyzing several metrics, we can conclude (1) that both the mean of the normalized GSR signal and the average acceleration of the surgical instrument are closely related to stress (i.e., they change significantly when presented with stressors) and (2) that the average acceleration was found to be correlated to the GSR, suggesting that there may be a link between psychomotor skills and personal resourcefulness. Further experiments are nevertheless required in order to extract further evidence from other metrics and tasks, together with an in-depth analysis of the influence of expertise.

Thus, the next steps, based on the results of this study, would be aimed at gathering information from surgeons with different specialties while performing a more complex laparoscopic task (such as suturing) in which stressors are included. For these new experiments, we aim at obtaining a larger number of participants in order to ensure higher statistical power. In addition to this, the differences between the obtained metrics in immersive simulated environments would also be of interest. We will also study the validity of the use of these metrics as a part of the assessment of personal resourcefulness as compared with validated scales (such as NOTECHS [99]). Furthermore, the study of a combination of metrics that are closely related to expertise could also contribute to the assessment of skills (both psychomotor and personal resourcefulness, simultaneously).

In conclusion, the results that we obtained demonstrate that it is feasible to use physiological monitoring and gaze analysis in order to obtain relevant information about the stress and attention levels of laparoscopic surgeons, which are directly related to their personal resourcefulness skills. This information could ultimately be used for the assessment of skills that are related to personal resourcefulness in surgical educational settings. Moreover, certain metrics that are related to personal resourcefulness have demonstrated to correlate with psychomotor skills metrics, which opens the way towards a complete objective assessment of surgical proficiency.

## Figures and Tables

**Table 1:** Metrics of stress derived from ECG and HRV. Where $t_{NN}{}_{i}$ denotes the time intervals between two consecutive R peaks, x_LF_ is the filtered version of the HRV signal in the frequency band [0.05–0.15] Hz, T is the period of the signal and x_HF_ is the filtered version of the HRV signal in the frequency band [0.16–0.50] Hz.

Metric	Description	Formula
Time-domain metrics		
AVNN	Average value of NN intervals (ms).	$AV_{NN}=\frac{1}{N}\sum_{i=1}^{N}t_{NN}{}_{i}$
SDNN	Standard deviation of NN intervals.	$SD_{NN}=\sqrt{\;\frac{1}{N}\sum_{i=1}^{N}{\left(t_{NN}{}_{i}-AV_{NN}\right)}^{2}}$
RMSSD	Root mean square of successive differences between successive NN intervals.	$RMS_{SD}=\sqrt{\;\frac{1}{N}\sum_{i=1}^{N}t_{NN}{{}_{i}}^{2}}$
PNN20	Ratio of the number of pairs of consecutive NN intervals differing by more than 20 ms over the total number of NN intervals.	$P_{NN}20=\frac{\left|\left\{i\;|\;t_{NN}{}_{i}>20ms\;\right\}\right|}{total\;No.\;of\;NN\;intervals}$
PNN50	Ratio of the number of pairs of consecutive NN intervals differing by more than 50 ms over the total number of NN intervals.	$P_{NN}50=\frac{\left|\left\{i\;|\;t_{NN}{}_{i}>50ms\;\right\}\right|}{total\;No.\;of\;NN\;intervals}$
Frequency-domain metrics		
LF	Absolute power of the signal in low frequency bands.	$LF=\frac{1}{T}\sum_{t=1}^{T}x_{LF}^{2}\left(t\right)$
HF	Absolute power of the signal in high frequency bands.	$LF=\frac{1}{T}\sum_{t=1}^{T}x_{HF}^{2}\left(t\right)$
LF-HF ratio	Ratio between the total energy in low frequency and the total energy in the high frequency.	$LF-HF\;ratio=\frac{\mathrm{LF}}{\mathrm{HF}}$
LFnu	Normalized spectral LF index.	${\mathrm{LF}\;}_{nu}=\frac{\mathrm{LF}}{\mathrm{LF}\;+\;\mathrm{HF}}$
HFnu	Normalized spectral HF index	${\mathrm{HF}\;}_{nu}=\frac{\mathrm{HF}}{\mathrm{LF}\;+\;\mathrm{HF}}$

**Table 2:** MAPs obtained from EVA tracking system.

MAPs.	Definition	Formulae
Time (T)	Total time to perform a task (s)	T
Path length (PL)	Total path covered by the instrument in the setting (m)	$PL=\int_{t=0}^{T}\frac{d\left|r\left(t\right)\right|}{dt}dt$
Average speed (S)	Rate of change of the instrument’s position in the setting (mm/s). Results are measured for the total magnitude and in each Cartesian direction of the box trainer.	$S_{u\left(t\right)}=\frac{1}{T}\int_{t=0}^{T}\frac{d\left|u\left(t\right)\right|}{dt}dt$
Average acceleration (A)	Rate of change of the instrument’s velocity within the setting (mm/s2)	$A=\frac{1}{T}\int_{t=0}^{T}\frac{d^{2}\left|r\left(t\right)\right|}{dt^{2}}dt$
Economy of area (EOA)	Relationship between maximum surface area (task plane) occupied by the instrument and total path length	$EOA=\frac{\sqrt{SU*}}{PL}$
Economy of volume (EOV)	Relationship between maximum volume occupied by the instrument in the setting and total path length	$EOA=\frac{\sqrt[{3}]{MV**}}{PL}$
Depth (D)	Total path length travelled in the instrument’s axis direction (m)	$D=\int_{t=0}^{T}\sqrt{{\frac{dy\left(t\right)}{dt}}^{2}+{\frac{dz\left(t\right)}{dt}}^{2}}dt$

* $SU=\left[max_{t}\left(x\left(t\right)\right)-min_{t}\left(x\left(t\right)\right)\right]\cdot \left[max_{t}\left(z\left(t\right)\right)-min_{t}\left(z\left(t\right)\right)\right]$ ** $MV=SU\cdot \left[max_{t}\left(y\left(t\right)\right)-min_{t}\left(y\left(t\right)\right)\right]$.

## Data Availability

Data available on request due to restrictions eg privacy or ethical.

## Supplementary Materials

The following supporting information can be downloaded at: https://www.mdpi.com/article/10.3390/s22030837/s1, Table S1. Summary of results of extracted metrics as related to stress. All technical metrics presented are from the right instrument (dominant hand). F(1,14) represents the F value of the test, being 1 and 14 the degrees of freedom of the test; U(8,8) represents the U test statistic, being 8 the sample size of subjects in the first and second phase; p indicates the *p*-value of the corresponding statistical test.
