# Supplementary material for: Correlating Personal Resourcefulness and Psychomotor Skills: An Analysis of Stress, Visual Attention and Technical Metrics
Source: Sensors (Basel). 2022 Jan 22;22(3):837. doi: 10.3390/s22030837 (PMC8838092; doi:10.3390/s22030837)
Supplement: Supplementary file 1 [file sensors-22-00837-s001.zip › sensors-1491215-supplementary.pdf]

Table S1. Summary of results of extracted metrics as related to stress. All technical metrics presented are from the right instrument (dominant hand). F(1,14) represents the F value of the test, being 1 and 14 the degrees of freedom of the test; U(8,8) represents the U test statistic, being 8 the sample size of subjects in the first and second phase; p indicates the *p*-value of the corresponding statistical test.

Metric	Phase 1	Phase 2	Test Results
**Psychomotor skills – Technical metrics**			
Time (mean ± SD)	146.03 ± 28.19	143.62 ± 20.36	F(1,14) = 0.038, *p* = 0. 907
Path length (mean ± SD)	2.97 ± 1.24	3.78 ± 1.04	F(1,14) = 2.018 *p* = 0.628
Average speed (mean ± SD)	21.18 ± 3.90	20.56 ± 3.41	F(1,14) = 0.113, *p* = 0.907
Average acceleration (median ± range)	0.06 ± 0.52	0.38 ± 1.06	U(8,8) = 10, *p* = 0.041*
Economy area (median ± range)	0.02 ± 0.07	0.02 ± 0.01	U(8,8) = 37, *p* = 0.642
Economy volume (median ± range)	0.03 ± 0.16	0.02 ± 0.01	U(8,8) = 50, *p* = 0.190
Depth (mean ± SD)	2.94 ± 1.2	3.73 ± 0.91	F(1,14) = 0.463, *p* = 0. 864
**Personal resourcefulness – Visual attention metrics**			
Ratio inside ROI (mean ± SD)	0.63 ± 0.22	0.65 ± 0.17	F(1,14) = 0.024, *p* = 0. 907
Average fixation duration (median ± range)	8.945 ± 0.7	8.93 ± 1.43	U(1,14) = 34, *p* = 0. 864
Fixation rate (mean ± SD)	0.54 ± 0.29	0.54 ± 0.24	F(1,14) = 0.001, *p* = 0.977
**Personal resourcefulness – Stress metrics**			
ECG mean (median ± range)	548.09 ± 25.34	549.19 ± 28.1	U(8,8) = 31, *p* = 0. 907
HR mean (mean ± SD)	90.65 ± 15.46	97.04 ± 13.22	F(1,14) = 0.791, *p* = 0.864
AVnn mean (mean ± SD)	676.62 ± 101.51	627.65 ± 79.02	F(1,4) = 1.133, *p* = 0.763
SDnn mean (median ± range)	51.79 ± 21.02	64.97 ± 34.68	U(8,8) = 23, *p* = 0.645
RMSsd mean (median ± range)	33.06 ± 8.67	37.74 ± 13.71	U(8,8) = 22, *p* = 0. 628
Pnn20 (mean ± SD)	0.28 ± 0.2	0.3 ± 0.19	F(1,14) = 0.289, *p* = 0.864
Pnn50 (mean ± SD)	0.08 ± 0.09	0.10 ± 0.08	F(1,14) = 0.241, *p* = 0.864
LF (median ± range)	1069.09 ± 717.99	1016.01 ± 808.96	U(8,8) = 33, *p* = 0. 907
HF (median ± range)	171.43 ± 103.77	246.17 ± 202.47	U(8,8) = 28, *p* = 0. 864
Ratio LF/HF (mean ± SD)	9.62 ± 6.22	8.28 ± 5.56	F(1,14) = 0.046, *p* = 0. 907
LF_{nu} (mean ± SD)	0.84 ± 0.12	0.8 ± 0.11	F(1,14) = 0.385, *p* = 0. 864
HF_{nu} (mean ± SD)	0.21 ± 0.19	0.27 ± 0.22	F(1,14) = 0.487, *p* = 0. 864
GSR mean (median ± range)	0.18 ± 0.45	0.66 ± 0.14	U(8,8) = 2, *p* = 0.002 *

* $p < 0.05$.
